# Hydrogen Sulfide Restored the Diurnal Variation in Cardiac Function of Aging Mice

**DOI:** 10.1155/2021/8841575

**Published:** 2021-03-02

**Authors:** Huaxing Zhang, Jing Dai, Danyang Tian, Lin Xiao, Hongmei Xue, Qi Guo, Xiangjian Zhang, Xu Teng, Sheng Jin, Yuming Wu

**Affiliations:** ^1^School of Basic Medical Sciences, Hebei Medical University, Hebei 050017, China; ^2^Department of Clinical Diagnostics, Hebei Medical University, Hebei 050017, China; ^3^Department of Physiology, Hebei Medical University, Hebei 050017, China; ^4^Hebei Collaborative Innovation Center for Cardio-Cerebrovascular Disease, 050017 Hebei, China

## Abstract

The present study was performed to investigate whether H_2_S could restore the diurnal variation in cardiac function of aging mice and explore the potential mechanisms. We found that ejection fraction (EF) and fractional shortening (FS) in 3-month-old mice exhibited diurnal variations over a 24-hour period. However, the diurnal variations were disrupted in 18-month-old mice, and there was a decline in EF and FS. In addition, the plasma malondialdehyde (MDA) levels were increased, and H_2_S concentrations and superoxide dismutase (SOD) activities were decreased in 18-month-old mice. Then, CSE KO mice were used to determine if there was a relationship between endogenous H_2_S and diurnal variations in EF and FS. There was no difference in 12-hour averaged EF and FS between dark and light periods in CSE KO mice accompanying increased MDA levels and decreased SOD activities in plasma, indicating that deficiency of endogenous H_2_S blunted diurnal variations of cardiac function. To determine whether oxidative stress disrupted the diurnal variations in cardiac function, D-galactose-induced subacute aging mice were employed. After 3-month D-gal treatment, both 12-hour averaged EF and FS in dark or light periods were decreased; meanwhile, there was no difference in 12-hour averaged EF and FS between dark and light periods. After 3-month NaHS treatment in the D-gal group, the plasma MDA levels were decreased and SOD activities were increased. The EF and FS were lower during the 12-hour light period than those during the 12-hour dark period which was fit to sine curves in the D-gal+NaHS group. Identical findings were also observed in 18-month-old mice. In conclusion, our studies revealed that the disrupted diurnal variation in cardiac function was associated with increased oxidative stress and decreased H_2_S levels in aging mice. H_2_S could restore the diurnal variation in cardiac function of aging mice by reducing oxidative stress.

## 1. Introduction

Because of the planet's rotation, life on earth has evolved strategies to better adapt its physiology and behaviors to diurnal cycles of around 24 h. In order to optimally match the dramatic fluctuations in physiological demands over the course of the day, the cardiac function peaks during the active phase and reaches a nadir in the rest phase, which is driven by a complex interaction between extrinsic (e.g., neurohumoral factors) and intrinsic (e.g., circadian clock) influences [1, 2]. The epidemiologic, clinical, and experimental studies demonstrate the profound importance of this 24 h circadian variation for healthy cardiovascular physiology, and disruption is associated with increased risk of heart disease and worsens outcome [3–5]. Furthermore, during various disease states (including aging), the circadian variation of cardiac function is often attenuated/abolished which is contributing to high morbidity and mortality of cardiovascular diseases in the elderly [6–8]. Several lines of evidence suggested that increasing oxidative stress was one of the main characteristics and drivers of aging and age-related disorders [9, 10]. It has been reported that there was a bidirectional relationship between oxidative stress and circadian rhythms as redox status could influence circadian clock function, while clock proteins could regulate redox homeostasis of cells [11, 12]. Increased oxidative stress may influence cardiac circadian genes and cardiac metabolism, which disrupted the diurnal variation of cardiac function [13]. Therefore, it is particularly important to determine the exact mechanisms underlying the role of aberrant circadian function in cardiac dysfunction of aging and explore new approaches for disease treatment/prevention.

Hydrogen sulfide (H_2_S), previously known only as toxic gas and environmental hazard, in the past 2 decades or so has been recognized as an important endogenous gasotransmitter similar to nitric oxide and carbon monoxide, since the landmark publication by Kimura in 1996 [14]. In mammals, H_2_S is endogenously generated, via the cysteine biosynthesis pathway, by three vital enzymes: cystathionine-*γ*-lyase (CSE), cystathionine-*β*-synthase (CBS), and 3-mercaptopyruvate sulfurtransferase (3-MST) [15]. CSE is the main enzyme for H_2_S production in the cardiovascular system, while CBS is mainly expressed in the central nervous system. 3-MST is located predominantly in the mitochondria and produces H_2_S in concert with cysteine aminotransferase (CAT). It has been demonstrated that physiological concentrations of H_2_S play important regulatory roles in the homeostasis of the cardiovascular system, and a decrease in the endogenous production of H_2_S has been associated with several age-related cardiovascular diseases such as hypertension, atherosclerosis, and heart failure [16–18]. Although the precise mechanism is still largely unknown, increasing evidence has shown that treatment with exogenous H_2_S prevents or reverses aging and age-related pathologies [19, 20]. In addition, our previous study found that plasma H_2_S concentration exhibited diurnal fluctuations [21]. And GYY4137, an exogenous H_2_S donor, or AOAA, the CBS inhibitor, also regulated the general expression and dynamics of several clock genes [22]. However, it is not yet clear whether disturbing diurnal variation in age-related cardiac dysfunction is associated with the changes in H_2_S concentration.

With this in mind, the aim of the present study was to investigate whether H_2_S may restore the diurnal variation in cardiac function of aging mice and explore the potential mechanisms.

## 2. Material and Methods

### 2.1. Animals and Treatments

Male C57BL/6J mice were purchased from Vital River Laboratories (Beijing, China). The CSE heterozygote mice with C57BL/6J genetic bases were kindly provided as gifts by Professor Yichun Zhu (Fudan University, Shanghai, China). CSE wild-type (WT) and knockout (CSE KO) mice were used in the experiments while heterozygote mice were maintained for breeding. Mice were housed in plastic cages in a room with a controlled humidity of 60%, at a temperature of 22-24°C and on a regular 12 h light and dark cycle (lights on from 8:00 to 20:00). They were fed on standard rat chow and tap water *ad libitum*.

Fifteen-month-old male C57BL/6 mice were randomly divided into 2 groups: old group and old+NaHS group. The mice in the old+NaHS group were intraperitoneally injected with NaHS (100 *μ*mol/kg/day) for 3 months, while the mice in the old group were injected with normal saline for the same period. Three-month-old male C57BL/6 mice were used in the control group.

In order to observe the effect of endogenous H_2_S, 8-week-old male CSE KO and WT mice were used for the experiments.

In addition, D-galactose (D-gal) was administered (125 mg/kg, subcutaneously) once daily for 3 months to establish the subacute aging model as previously described with some modified [23], and the D-gal treatment mice were randomly divided into 2 groups: D-gal group and D-gal+NaHS group. The mice in the D-gal+NaHS group were intraperitoneally injected with NaHS (100 *μ*mol/kg/day) for 3 months, while the mice in the D-gal group were injected with normal saline for the same period.

At the end of the experiment, the cardiac function was evaluated by echocardiography every four hours (8:00, 12:00, 16:00, 20:00, 24:00, and 8:00). And then, mice were euthanized with 3% isoflurane, and blood was collected. Plasma was separated from the blood after centrifugation at 3500 rpm for 10 min and then stored at -80°C until assay.

All our animal experimental procedures were performed according to the *Guide for the Care and Use of Laboratory Animals* of the National Institutes of Health (NIH) of the United States and approved by the Ethics Committee for Laboratory Animals Care and Use of Hebei Medical University.

### 2.2. Echocardiography

To evaluate left ventricular function, mouse two-dimensional echocardiography was performed using a Vevo 2100 ultrasound device (FUJIFILM VisualSonics Inc., Toronto, Canada). Mice were anaesthetized with 1% isoflurane, and M-mode images of the left ventricle were recorded. All measurements were averaged for five consecutive cardiac cycles. Left ventricular ejection fraction and fractional shortening (LVEF and LVFS) were measured to evaluate cardiac function.

### 2.3. Measurement of H_2_S Concentration in Plasma

The H_2_S in plasma was measured according to previously described methods [24]. Thirty microliters of plasma was mixed with 80 *μ*L MBB and 10 *μ*L 0.1% ammonia with shaking for 1 h at room temperature for derivatization of sulfide. MBB reacts with sulfide to produce sulfide-dibimane (SDB). SDB is more hydrophobic than most physiological thiols and can be separated by gradient elution and analyzed by liquid chromatography-tandem mass spectrometry. The reaction was then terminated with 10 *μ*L 20% formic acid and centrifuged at 15000 g for 10 min. The supernatants were stored at -80°C until H_2_S measurements were done. H_2_S concentrations were determined by using a curve generated with sodium sulfide (0-40 *μ*mol/L) standards.

### 2.4. Measurement of Malondialdehyde (MDA) Concentration in Plasma

The MDA concentration in plasma was determined by using a commercial assay kit (Beyotime Biotechnology Co., Ltd., China) according to the manufacturer's instructions.

### 2.5. Measurement of Superoxide Dismutase (SOD) Activity in Plasma

The activity of SOD in plasma was determined by using a commercial assay kit (Jiancheng Bioengineering Institute, China) according to the manufacturer's instructions.

### 2.6. Statistical Analysis

Results were expressed as the mean ± SEM. Statistical analysis was performed using an SPSS software package, version 13.0 (SPSS, Inc., Chicago, USA). Comparisons between two groups were made using an independent *t*-test. *P* < 0.05 was considered statistically significant.

## 3. Results

### 3.1. Diurnal Variations in EF and FS Were Disrupted in Aging Mice

**Figure 1:**
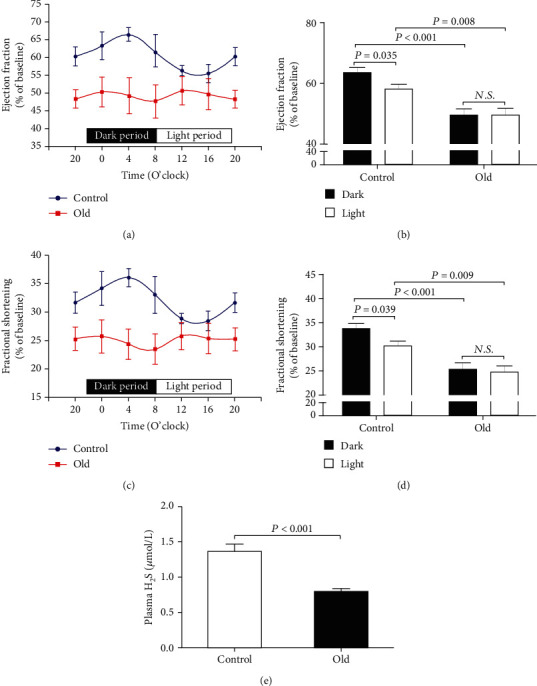
Diurnal variations in EF and FS were disrupted in aging mice. (a) Diurnal variation of EF in 18-month-old mice. (b) Mean value of EF during the 12-hour light or dark period in 18-month-old mice. (c) Diurnal variation of FS in 18-month-old mice. (d) Mean value of FS during the 12-hour light or dark period in 18-month-old mice. (e) Plasma H_2_S levels in 18-month-old mice. Results are means ± SEM. A *P* of <0.05 was considered significant.

As was shown in Figures 1(a) and 1(c), EF and FS, the indicators of cardiac function measured by echocardiography, exhibited diurnal variations over a 24-hour period in 3-month-old control mice. The 4-hour interval data showed that both EF and FS were significantly lower during the 12-hour light (inactive) period than those during the 12-hour dark (active) period (Figures 1(b) and 1(d)), and the minimum and maximum values were observed at 16:00 and 4:00, respectively, which were fit to sine curves. However, both EF and FS in 18-month-old mice were comparable between the 12-hour light period and the 12-hour dark period, indicating that diurnal variations in EF and FS were disrupted in aging mice. Meanwhile, both EF and FS in 18-month-old mice were significantly lower than those in 3-month-old control mice at any time point during the 4-hour interval. In addition, the plasma H_2_S concentrations in old mice were significantly lower than those in control mice (Figure 1(e)).

### 3.2. Diurnal Variations in EF and FS Were Blunted in CSE KO Mice

**Figure 2:**
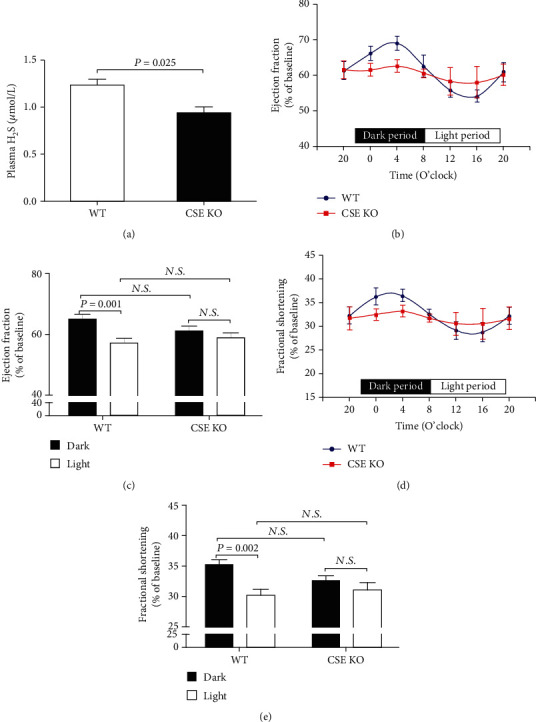
Diurnal variations in EF and FS were blunted in CSE KO mice. (a) Plasma H_2_S levels in CSE KO mice. (b) Diurnal variation of EF in CSE KO mice. (c) Mean value of EF during the 12-hour light or dark period in CSE KO mice. (d) Diurnal variation of FS in CSE KO mice. (e) Mean value of FS during the 12-hour light or dark period in CSE KO mice. Results are expressed as the mean ± SEM. A *P* of <0.05 was considered significant.

CSE was the main enzyme for H_2_S production in the cardiovascular system, so 8-week-old CSE KO mice were used to determine if there was a relationship between endogenous H_2_S and diurnal variations in EF and FS. As was shown in Figure 2(a), H_2_S concentrations were significantly decreased in CSE KO mice as compared with those in WT mice. Although the maximum and minimum values of EF and FS still were observed at 4:00 and 16:00, respectively (Figures 2(b) and 2(d)), there was no difference in 12-hour averaged EF and FS between dark and light periods in CSE KO mice (Figures 2(c) and 2(e)). These results indicated that diurnal variations in EF and FS were blunted in CSE KO mice.

### 3.3. Disruption of Diurnal Variations in EF and FS Was Induced by Oxidative Stress

**Figure 3:**
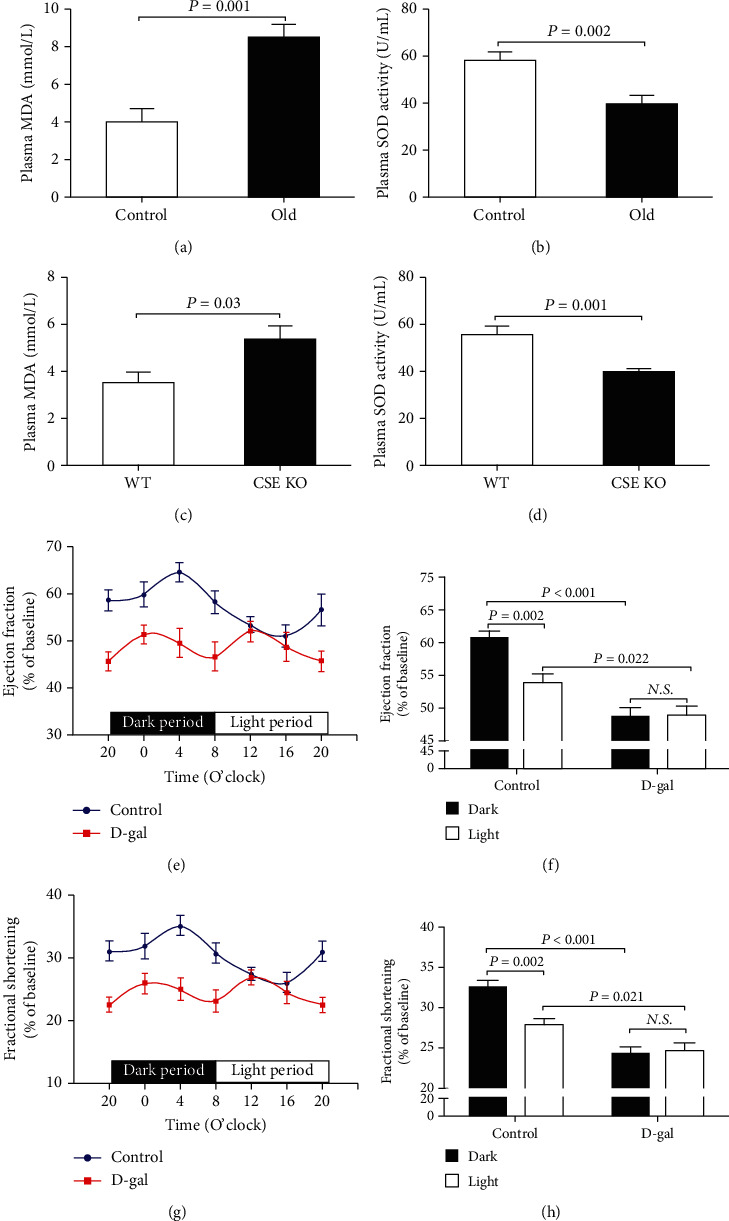
Disruption of diurnal variations in EF and FS was induced by oxidative stress. (a) Plasma MDA levels in 18-month-old mice. (b) Plasma SOD activities in 18-month-old mice. (c) Plasma MDA levels in CSE KO mice. (d) Plasma SOD activities in CSE KO mice. (e) Diurnal variation of EF in D-gal-induced subacute aging mice. (f) Mean value of EF during the 12-hour light or dark period in D-gal-induced subacute aging mice. (g) Diurnal variation of FS in D-gal-induced subacute aging mice. (h) Mean value of FS during the 12-hour light or dark period in D-gal-induced subacute aging mice. Results are expressed as the mean ± SEM. A *P* of <0.05 was considered significant.

Because oxidative stress was considered a key mechanism mediating age-related diseases, the contents of MDA, a lipid oxidation final product, and the activities of SOD, a main antioxidant enzyme in plasma, were detected to reflect oxidative stress levels in the aging and CSE KO mice. As was shown in Figures 3(a) and 3(b), compared with the 3-month-old control mice, the plasma MDA levels were significantly increased and SOD activities were significantly decreased in 18-month-old mice. Similar changes were also observed in CSE KO mice as compared with WT mice (Figures 3(c) and 3(d)). To determine whether oxidative stress disrupted the diurnal variations in EF and FS, D-gal-induced subacute aging mice were employed. As was shown in Figures 3(e)–3(h), both 12-hour averaged EF and FS in dark or light periods were significantly lower in the D-gal group than those in the control group; meanwhile, there was no difference in 12-hour averaged EF and FS between dark and light periods in D-gal-induced subacute aging mice.

### 3.4. H_2_S Restored the Diurnal Variations of EF and FS in Subacute Aging Mice

**Figure 4:**
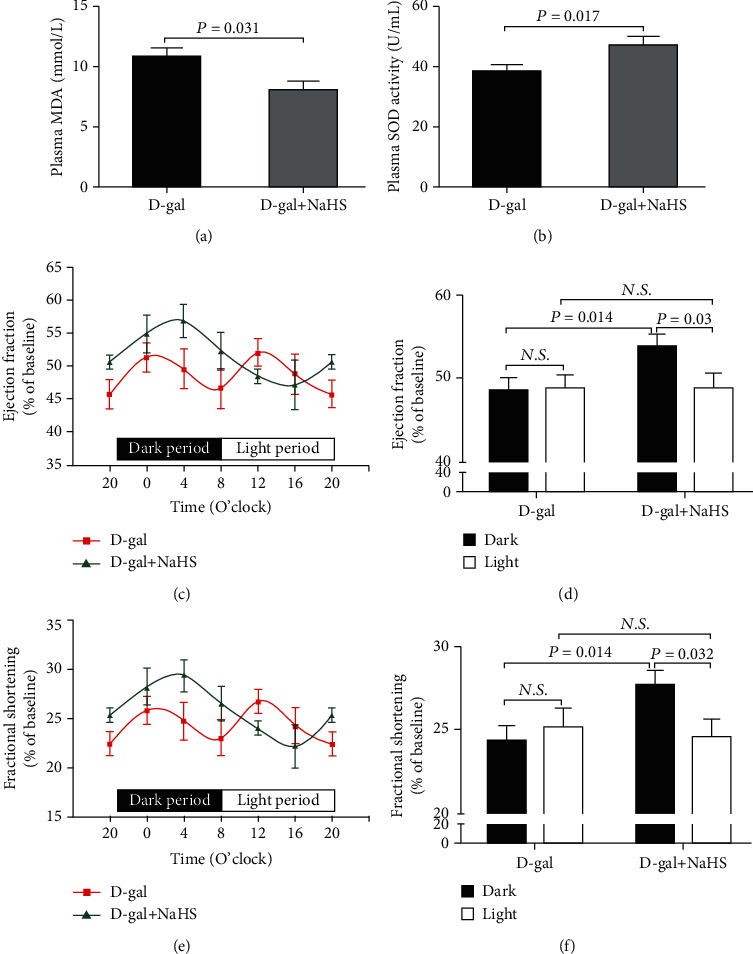
H_2_S restored the diurnal variations of EF and FS in subacute aging mice. (a) Plasma MDA levels in D-gal-induced subacute aging mice after NaHS treatment. (b) Plasma SOD activities in D-gal-induced subacute aging mice after NaHS treatment. (c) Diurnal variation of EF in D-gal-induced subacute aging mice after NaHS treatment. (d) Mean value of EF during the 12-hour light or dark period in D-gal-induced subacute aging mice after NaHS treatment. (e) Diurnal variation of FS in D-gal-induced subacute aging mice after NaHS treatment. (f) Mean value of FS during the 12-hour light or dark period in D-gal-induced subacute aging mice after NaHS treatment. Results are expressed as the mean ± SEM. A *P* of <0.05 was considered significant.

As was shown in Figures 4(a) and 4(b), compared with the D-gal group, the plasma MDA levels were significantly decreased and SOD activities were significantly increased in the D-gal+NaHS group. In the D-gal+NaHS group, both EF and FS were significantly lower during the 12-hour light (inactive) period than those during the 12-hour dark (active) period (Figures 4(d) and 4(f)), and the minimum and maximum values were observed at 16:00 and 4:00, respectively, which were fit to sine curves (Figures 4(c) and 4(e)). Although there was no difference in 12-hour averaged EF and FS in the light period between the two groups, the 12-hour averaged EF and FS in the dark period were significantly higher in the D-gal+NaHS group than those in the D-gal group.

### 3.5. H_2_S Restored the Diurnal Variations of EF and FS in Aging Mice

**Figure 5:**
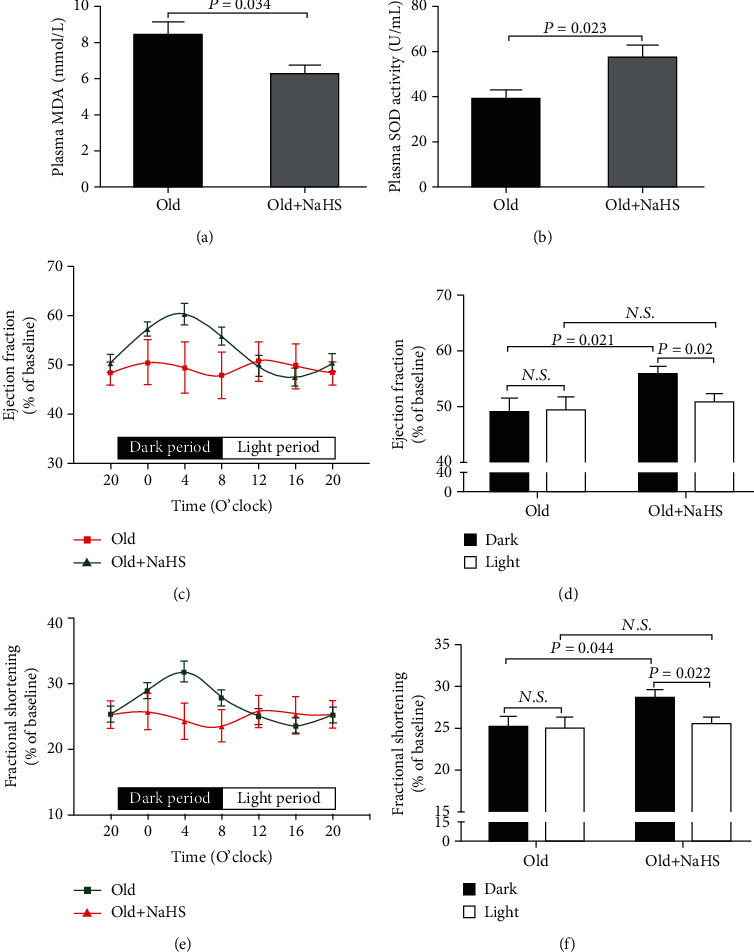
H_2_S restored the diurnal variations of EF and FS in aging mice. (a) Plasma MDA levels in 18-month-old mice after NaHS treatment. (b) Plasma SOD activities in 18-month-old mice after NaHS treatment. (c) Diurnal variation of EF in 18-month-old mice after NaHS treatment. (d) Mean value of EF during the 12-hour light or dark period in 18-month-old mice after NaHS treatment. (e) Diurnal variation of FS in 18-month-old mice after NaHS treatment. (f) Mean value of FS during the 12-hour light or dark period in 18-month-old mice after NaHS treatment. Results are expressed as the mean ± SEM. A *P* of <0.05 was considered significant.

As was shown in Figure 5, NaHS treatment in 18-month old mice caused similar changes with subacute aging mice. After 3-month NaHS treatment, the plasma MDA levels were significantly decreased and SOD activities were significantly increased (Figures 5(a) and 5(b)); meanwhile, both EF and FS were significantly lower during the 12-hour light (inactive) period than those during the 12-hour dark (active) period and showed significant fits to sine curves (Figures 5(c)–5(f)).

## 4. Discussion

In the present study, we found that (i) there was a decreased EF, FS, and robustness of diurnal variation in aging mice which was induced by increased oxidative stress and decreased hydrogen sulfide levels in the aging process; (ii) three-month H_2_S treatment could restore the diurnal variation in cardiac function of aging mice by reducing oxidative stress.

In order to anticipate and adapt to the diurnal environmental changes, various physiological, metabolic, and behavioral processes in organisms exhibit a 24 h circadian rhythm, which is particularly prominent in the cardiovascular system. Cardiovascular physiology including blood pressure, heart rate, and cardiac contractility all peak in the wake hours and reach a nadir during sleep [25, 26]. The onset of cardiovascular diseases (CVD_S_), such as myocardial infarction and sudden cardiac death, also follows a diurnal pattern, and disruption of rhythmicity is associated with increased risk of CVD_S_ and adverse health consequences. These daily fluctuations in cardiovascular functions are driven by both rhythmic neurohormonal regulation derived from the central clock in the suprachiasmatic nucleus and rhythmic gene expression originating from the peripheral clock in different organs. The absence of clock genes results in a loss of circadian rhythms, an acceleration of aging, and a shortened life span in mice [27, 28]. Similarly, with advanced age, the clock genes become disorganized which leads to the reduction in the amplitude or even abolition of the rhythms. In the present study, EF and FS in 3-month-old mice exhibited diurnal variations over a 24-hour period. Both EF and FS were significantly lower during the 12-hour light period than those during the 12-hour dark period, and the minimum and maximum values were observed at 16:00 and 4:00, respectively. However, diurnal variations in EF and FS were disrupted in 18-month-old mice. Moreover, there was a decline in EF and FS in old mice than those in control mice at any time point during the 4-hour interval. Our results suggested that aging not only impaired cardiac function but also led to disruption of the circadian rhythms in the heart. These results were congruent with Wang et al. [29] who found that aging disrupted normal time-of-day variation in cardiac electrophysiology, Ca^2+^ handling, and adrenergic responsiveness in the isolated adult hearts. It was also reported that age-associated changes in intrinsic clock mechanisms within sinoatrial node cells blunted circadian rhythms in the heart rate [30].

Although the exact mechanisms remain unclear, it is considered that imbalance of redox homeostasis which manifests as enhanced reactive oxygen species (ROS) and their by-product production and decreased ROS-scavenging enzymes such as SOD and catalase plays a central role in the process of aging [31]. In the present study, the plasma MDA levels, a lipid oxidation final product, were significantly increased, and SOD activities, a main antioxidant enzyme, were significantly decreased in 18-month-old mice, which was in line with several other studies [32, 33]. A number of studies suggested that the redox status and levels of endogenous antioxidants were circadian regulated and varied in a time-of-day-dependent manner [34, 35], while, apart from some pioneering studies [11, 36], remarkably little was known whether oxidative stress modulates circadian rhythms, particularly in cardiac function. To determine whether oxidative stress disrupted the diurnal variations in cardiac function, D-gal-induced subacute aging mice were employed in the present study. D-gal administration gave rise to the generation of ROS causing oxidative stress injury similar in many ways to human aging and had been extensively applied to study the mechanisms of aging [37]. After 3-month D-gal treatment, both 12-hour averaged EF and FS in dark or light periods were significantly decreased; meanwhile, there was no difference in 12-hour averaged EF and FS between dark and light periods, which indicated that oxidative stress led to disruption of the circadian rhythms in the heart.

H_2_S which is endogenously generated by CSE, CBS, and 3-MST functions as a signaling molecule in an array of physiological processes. Interestingly, as a radical scavenger, H_2_S has been recently recognized as a new player capable of influencing oxidative stress involved in aging, and then, it is viewed as a potential target for preventing aging. H_2_S can reduce oxidative stress by quenching free radicals as a chemical reductant, scavenging free radicals via nonenzymatic antioxidants or via enzymatic antioxidants, and inhibiting the mitochondrial free radical production [38]. It has been demonstrated that the decrease in the endogenous production of H_2_S has been associated with aging and oxidative stress injuries [18, 39–41]. It also was found that H_2_S enhanced the expression or activity of NAD-dependent deacetylase sirtuin-1 to delay cell senescence or maintain the circadian rhythm of the clock gene [42, 43]. In addition, as nitric oxide which had daily rhythms [44], our previous study found that plasma H_2_S concentrations exhibited diurnal fluctuations [21]. In the present study, the plasma H_2_S concentrations in 18-month-old mice were significantly decreased which might be associated with the disruption of diurnal variations in cardiac function. CSE was the main enzyme for H_2_S production in the cardiovascular system, so CSE KO mice were used to determine if there was a relationship between endogenous H_2_S and diurnal variations in cardiac function. As previously described [45], plasma H_2_S was significantly decreased, and there was no difference in 12-hour averaged EF and FS between dark and light periods in CSE KO mice. In addition, compared with WT mice, the plasma MDA levels were significantly increased and SOD activities were significantly decreased in CSE KO mice which were in agreement with a previous study [46]. These results indicated that deficiency of endogenous H_2_S induced oxidative stress and blunted diurnal variations of cardiac function. In subsequent experiments, the D-gal-induced subacute aging mice or 15-month-old mice were intraperitoneally injected with NaHS, a H_2_S donor, for 3 months. NaHS treatment significantly reduced oxidative stress injury by increasing SOD activities and decreasing MDA levels both in subacute aging mice and normal aging mice. Meanwhile, after NaHS treatment, EF and FS were significantly lower during the 12-hour light period than those during the 12-hour dark period which was fit to sine curves both in subacute aging mice and normal aging mice. Our results indicated that 3-month H_2_S treatment restored the diurnal variations of cardiac function in aging mice, but more specific mechanisms needed further research.

Several limitations of the present study should be noted. Firstly, we only found decreased H_2_S levels, and increased oxidative stress was associated with the disrupted diurnal variation in cardiac function of aging mice. Although NaHS treatment significantly reduced MDA levels and increased SOD activities both in subacute aging mice and normal aging mice, there was no direct evidence provided on the relation of lower endogenous H_2_S levels and oxidative stress, something which would need further investigation in future studies. Secondly, the diurnal variations in H_2_S levels and related synthetic and metabolic enzymes were unknown, and more experiments needed to be performed to obtain more powerful results. Thirdly, the underlying mechanism of H_2_S treatment restoring the diurnal variations of cardiac function in aging mice was not explored in detail, by which future attention would be focused on.

In conclusion, the disrupted diurnal variation in cardiac function was associated with increased oxidative stress and decreased H_2_S levels in aging mice. H_2_S could restore the diurnal variation in cardiac function of aging mice by reducing oxidative stress.

## Data Availability

All data supporting the findings of this study can be available from the corresponding authors upon reasonable request.
